# Powerful inner/outer controlled multi-target magnetic nanoparticle drug carrier prepared by liquid photo-immobilization

**DOI:** 10.1038/srep04990

**Published:** 2014-05-21

**Authors:** Yan-Qing Guan, Zhe Zheng, Zheng Huang, Zhibin Li, Shuiqin Niu, Jun-Ming Liu

**Affiliations:** 1 Institute for Advanced Materials and School of Life Science, South China Normal University, Guangzhou 510631, China; 2 Laboratory of Solid State Microstructures, Nanjing University, Nanjing 210093, China; 3 Institute for Advanced Materials and Laboratory of Quantum Engineering and Quantum Materials, South China Normal University, Guangzhou 510631, China; 4 These authors contributed equally to this work.

## Abstract

Nanomagnetic materials offer exciting avenues for advancing cancer therapies. Most researches have focused on efficient delivery of drugs in the body by incorporating various drug molecules onto the surface of nanomagnetic particles. The challenge is how to synthesize low toxic nanocarriers with multi-target drug loading. The cancer cell death mechanisms associated with those nanocarriers remain unclear either. Following the cell biology mechanisms, we develop a liquid photo-immobilization approach to attach doxorubicin, folic acid, tumor necrosis factor-α, and interferon-γ onto the oleic acid molecules coated Fe_3_O_4_ magnetic nanoparticles to prepare a kind of novel inner/outer controlled multi-target magnetic nanoparticle drug carrier. In this work, this approach is demonstrated by a variety of structural and biomedical characterizations, addressing the anti-cancer effects *in vivo* and *in vitro* on the HeLa, and it is highly efficient and powerful in treating cancer cells in a valuable programmed cell death mechanism for overcoming drug resistance.

Magnetic nanoparticles (MNPs) are emerging as promising candidates for applications in biomedical research encompassing drug delivery, due to their small size, biocompatibility, and superparamagnetic behaviors^1^,^2^,^3^,^4^,^5^,^6^,^7^,^8^. Various groups usually use long-chain polymer oleic acid (OA) and its salts to stabilize iron oxide nanoparticles from aggregation and for the effective drug delivery^9^,^10^,^11^,^12^. In this approach, pharmaceutical drugs are loaded onto the surface of OA-MNPs, and then released at target sites in response to external stimuli, thereby offering a possibility of low toxicity but more accurately targeted dose. However, substantial effort in synthesizing OA-MNPs with effective pharmaceutical drugs is still needed before any clinical applications, in terms of the coating strategy, application dose, toxicology evaluation, metabolism mechanism *in vivo*, and then cell biology mechanism *in vitro*.

Various kinds of surface functionalization methodologies can be chosen to prepare multi-target MNPs with end groups including isocyanine, acrylate, thiol, amino, and carboxylic groups. Among them, the coating strategy of anti-cancer pharmaceutical drugs is one of the most active research areas^13^. Various MNPs functionalized with folic acid (FOL) can specifically promote the cancer cellular uptake through folate receptor mediated endocytosis. Many researches concentrated on potent folate conjugates with different drug mechanisms, novel linkers, and cleavable bonds^14^,^15^,^16^,^17^,^18^. Also, doxorubicin (DOX) is an effective and widely used chemotherapeutic agent associated with multi-target MNPs against quite a few of cancers^19^,^20^,^21^,^22^,^23^. It is noted that nuclear DNA is the main subcellular site of DOX action (intercalate DNA as a cytostatic and apoptotic agent) for its antitumor activity^24^,^25^.

Although a variety of methodologies have been developed to coat drug molecules onto multi-target MNPs, a practically accessible technique remains particularly challenging. One of the reasons is associated with the chemical structures and functional groups of these drugs that can be very different from one and another, and the quantity and locations of these functional groups are also structure-dependent. Therefore, it is difficult to covalently immobilize different drug molecules on the multi-target MNPs using the same method. In fact, coating of drug molecules onto OA-MNPs using a combination of various techniques will lead to tremendous difficulty in the preparation sequence^26^. Along this line, photo-immobilization can be an alternative approach to prepare such multi-target MNPs, because it has been used to immobilize various peptides, enzymes, growth factors, and proteins onto polymer surfaces in an effort to produce biomaterials with different activities and utilizations^27^,^28^,^29^,^30^,^31^.

On the other hand, Interferon-γ (IFN-γ) is an important cytokine which can enhance the antitumor effects of antimetabolite on cancer cells and induce or modulate apoptosis either as a single agent or in combination with tumor necrosis factor-α (TNF-α), which is another kind of cytokine produced by activated monocytes and phagocytes that could effectively kill many sorts of cancer cells both *in vivo* and *in vitro*^32^,^33^,^34^,^35^. In our earlier works, co-immobilized TNF-α plus IFN-γ was prepared by this approach to synthesize a series of polymeric anti-cancer drugs. The results revealed that the co-immobilized TNF-α plus IFN-γ can translate apoptosis signal for longer time than the free one, and does cause the activation of receptor protein IFNR2 and mainly induce caspase-independent PCD in human cervical cancer cells HeLa^36^,^37^,^38^,^39^. These experiences motivate us to argue that OA-MNPs with photo-immobilized DOX, FOL, TNF-α plus IFN-γ could remarkably enhance the inhibition to human cervical cancers.

This photo-immobilization approach, if working efficiently, would overcome the difficulty of covalent immobilization of various types of drug molecules (DOX, FOL, TNF-α, and IFN-γ). Since radical reactions occur on every organic material, including drug molecules and OA molecules coated on the surface of Fe_3_O_4_ nanoparticles, the photo-immobilization requires no special functional groups such as amino, carboxyl, hydroxyl, or thiol groups. In this work, we intend to develop a kind of multi-target MNPs which are more efficient in selective delivery of drug molecules to HeLa cells, assuring both cellular inner (DOX) and outer membrane (TNF-α plus IFN-γ) control as means to enhance the anti-cancer efficacy against HeLa cells, by photo-immobilization method.

Nevertheless, conventional photo-immobilization shows substantial drawbacks in synthesis of such multi-target MNPs. On one hand, for a layer of MNPs mixed with those drug molecules, the photo-immobilization can only occur for those particles and drug molecules on the top surface (limited thickness) exposed to the UV irradiation, while those underneath particles and drugs remain free of the UV irradiation. Although external stimuli such as mechanical vibration or stirring may partially avoid this problem, the low UV irradiation efficiency becomes obvious and no high drug loading is possible. On the other hand, although the MNPs are coated with oleic acid, the particle aggregation issue becomes inevitable in the immobilization process.

Therefore, the major concern of this work is to perform the photo-immobilization method in the preparation of multi-target magnetic nanoparticle drug carrier and even develop a method absent from those disadvantages and we call it the liquid photo-immobilization (LPI), while the conventional method is given a terminology of the solid photo-immobilization (SPI). More importantly, we demonstrate the successful immobilization of a series of anti-cancer drug molecules onto the surface of the oleic acid coated MNPs (OA-MNPs) using this LPI method, so as to synthesize a powerful inner/outer controlled multi-target MNP drug carrier in terms of not only the high drug loading and low toxicity, but also high anti-cancer activity with novel and valuable mechanism on HeLa.

## Results

### Chemical/physical preparation

**Figure 1:**
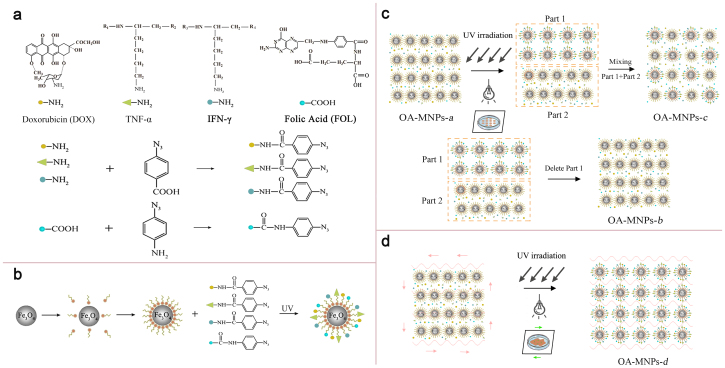
The molecular structures of the four kinds of photoactive molecules and the chemical processes for preparing photoactive DOX, FOL, TNF-α, and IFN-γ (a), general procedure of photo-immobilization sequence for multi-target MNPs (b), the corresponding nano-particles and sketch of photo-immobilization apparatus in the solid (upper, OA-MNPs-*a*, OA-MNPs-*b*, and OA-MNPs-*c*) (c) and liquid (lower, OA-MNPs-*d*) photo-immobilizations (d).

A schematic drawing of the condensed formulas of these drug molecules (DOX, FOL, TNF-α, IFN-γ) and chemical processes of photoreaction is given in Fig. 1a. The general procedure for photo-immobilizing these drugs with the OA-MNPs is presented in Fig. 1b, while Figs. 1c and 1d show the SPI (upper) and LPI (lower) schemes.

It is noted that for the SPI, only those particles on the surface layer (limited thickness) can chemically react with the surrounding drug molecules via the photo-grating process, since the UV-irradiation can not penetrate through the surface layer. For the LPI, when the drug molecules and OA-MNPs are dispersed in convective liquid solution in a container, all the OA-MNPs are well separated and thus can sufficiently react with the surrounding drug molecules upon the photo-grafting. Surely, the solution itself will absorb partially the UV-irradiation, however, a proper prolongation of the irradiation time would allow sufficient and homogeneous immobilization of those drug molecules onto particle surfaces at relatively high load. This efficiency is hardly achievable in the SPI, and therefore one can reasonably expect that this LPI scheme would be highly advantageous in synthesizing multi-target MNPs with high drug loading, homogeneity, and improved biomedical consequences.

A detailed description of the photo-immobilization optimization can be found in the Supplementary Information. We prepare four kinds of multi-target MNPs under the optimized conditions. They are OA-MNPs-(*a*, *b*, *c*, and *d*). The OA-MNPs-*a* are prepared by mixing the OA-coated particles with photoactive DOX, FOL, TNF-α, and IFN-γ without any UV-irradiation (Fig. 1c). We spread the OA-MNPs-*a* onto two plates and the OA-MNPs layer on each plate is ~1 mm thick. These OA-MNPs-*a* are submitted to the UV irradiation for 10 min using the SPI scheme. The as-prepared powder taken from one plate is the OA-MNPs-*c*, noting the particles on the top surface are immobilized with the drug molecules and those particles underneath are not. We remove the top surface powder on the other plate, and the remained underneath powder is the OA-MNPs-*b*, noting that the powder is similar to the OA-MNPs-*a*. The OA-MNPs-*d* are prepared by pouring the OA-coated particles and photoactive DOX, FOL, TNF-α, and IFN-γ into the convective liquid solution in a silica container which is submitted to the UV irradiation using the LPI scheme for 40 min. Subsequently, a series of characterizations on the four kinds of OA-MNPs are performed in order to demonstrate the advantages of the LPI scheme. Particular attention is paid to the OA-MNPs-*c* and -*d* for comparing their structures and biomedical performances.

### Morphology and size distribution

**Figure 2:**
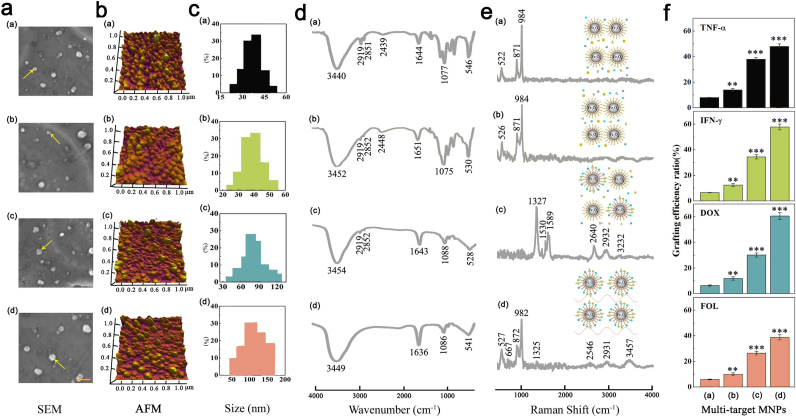
SEM images (a), AFM micrographs (x, y-scale of 1 μm) (b), size distribution (c), FTIR spectra (d), Raman spectra (e), and Grafting ratios (f) for the four kinds of OA-MNPs: OA-MNPs-*a*, OA-MNPs-*b*, OA-MNPs-*c*, and OA-MNPs-*d*. The yellow bar in the SEM images stands for 150 nm.

We first check the morphology and size distribution of the four kinds of OA-MNPs. Fig. 2a and 2b show the SEM and AFM images of OA-MNPs-*a* (a), -*b* (b), -*c* (c), and –*d* (d). The corresponding size distributions evaluated from the dynamic light scattering data are presented in Figs. 2c (a)–(d), respectively. All the four kinds of OA-MNPs are round-shaped in morphology and homogeneous in distribution. The OA-MNPs-*a* and -*b* are similar in size (~36.88 nm and ~39.86 nm), indicating no many drug molecules immobilized onto the particle surface. The OA-MNPs-*c* are much bigger (~81.36 nm), suggesting that these particles are successfully coated with the drug molecules. The OA-MNPs-*d* show the biggest size (~110.8 nm), suggesting much more drug loading and higher efficiency in the LPI than those in the SPI.

### Chemical bonding

Subsequently, we address the chemical bonding between the functional groups of the drug molecules and particle surface upon the photo-immobilization, using the Fourier transform infrared spectroscopy (FTIR). The FTIR data for the four kinds of OA-MNPs are summarized in Figs. 2d (a)–(d). For the OA-MNPs-*a*, -*b*, and -*c*, one observes characteristic peaks from the methyl (~2837 cm^−1^) and methylene (~2903 cm^−1^). For the OA-MNPs-*a* and -*b*, the two peaks can be reasonably assigned because the mixture of particles and drug molecules remains inactive (free of UV-irradiation). The appearance of the two peaks for the OA-MNPs-*c* implies that the SPI scheme is far from complete, since the azido groups from the photoactive DOX, FOL, TNF-α, and IFN-γ are very active under UV-irradiation and they will change to nitrogen radicals and capture hydrogen from any carbon to form the C-N covalent bonds, leading to the disappearance of methyl and methylene. No such peaks exist for the OA-MNPs-*d* when the LPI time is at least 40 min. This demonstrates sufficient immobilization reaction of the drug molecules with particles for the OA-MNPs-*d*. The fingerprint region (1500−400 cm^−1^) in the FTIR spectra shows tremendous variations for the OA-MNPs-*a* and -*b* in comparison with the OA-MNPs-*c* and -*d*, indicating that the drug molecules are successfully immobilized on the particle surfaces for the latter two cases.

### Chemical configuration and molecular skeleton

Additional evidence associated with the chemical configuration and molecular skeleton comes from the Raman spectroscopy of the four kinds of MNPs, as shown in Fig. 2e. The four peaks (~2640 cm^−1^, ~2932 cm^−1^, ~3232 cm^−1^, and ~3457 cm^−1^) are sensitive in response to the photo-grating. In addition, the abnormal appearance of peak from the methylene vibration (~1327 cm^−1^) and that from the double carbon bonds of the phenyl rings (~1589 cm^−1^) in the molecular skeleton reflects the significant configuration variation of the drug molecules in response to the SPI. At the same time, the three peaks (~526 cm^−1^, ~871 cm^−1^, and ~984 cm^−1^) arising from the loop breathing are available in the OA-MNPs-*a*, -*b*, and -*d*, but not in the OA-MNPs-*c*, indicating that the LPI does not break the chemical configuration of the drug molecules, but so does the SPI. A destruction of this conformation may damage the functionality of these drug molecules. Therefore, the LPI is also superior to the SPI in this sense.

### Grafting ratios

We check the grafting ratios of TNF-α, IFN-γ, DOX, and FOL for the four kinds of OA-MNPs. These data are summarized in Fig. 2f. For the OA-MNPs-*a*, -*b*, and -*c*, the grafting ratios of TNF-α, IFN-γ, DOX, and FOL are 8%, 6.5%, 6.4%, and 5.9%; 14%, 12.4%, 11.9%, and 10%; 38%, 34.5%, 30.2%, and 26.5%; in order for each case respectively. However, the OA-MNPs-*d* shows the highest grafting ratio, i.e. 48%, 57.8%, 60.6%, and 38.9%, further demonstrating the much more drug loading and higher efficiency in the LPI than those in the SPI.

### Activities and toxicities *in vivo*

The most convincing evidence supporting the LPI comes from the biomedical characterizations. We evaluate the efficacy of these multi-target MNPs using xenograft models by injecting HeLa cells into the flank of nude mice. After the tumors developed up to ~3000 mm^3^, we performed comparative efficacy studies by dividing animals into four groups (n = 6) in a way to minimize weight and tumor size differences among the groups. Using the multi-target MNPs at a dose of 1000 ng, which include 10 ng DOX, 10 ng FA, 10 ng TNF-α, and 10 ng IFN-γ at each time, the following groups were administered by injections every other day using: (a) the OA-MNPs-*a* (control group), (b) the OA-MNPs-*b*, (c) the OA-MNPs-*c*, and (d) the OA-MNPs-*d*.

**Figure 3:**
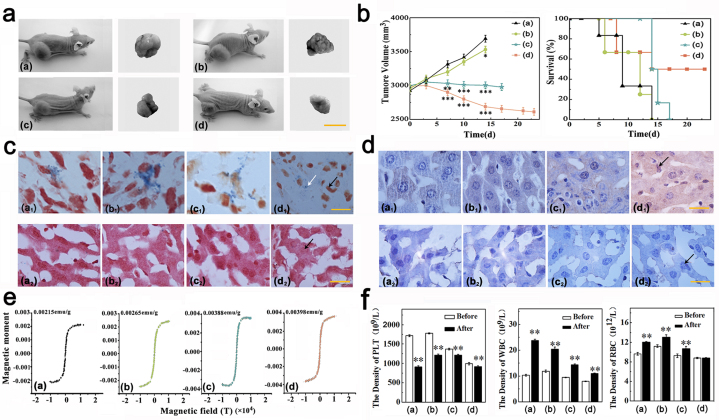
Measured efficacy and toxicities data *in vivo*. (a). Photos for tumor-bearing mice treated by the four kinds of OA-MNPs: (a) OA-MNPs-*a*, (b) OA-MNPs-*b*, (c) OA-MNPs-*c*, and (d) OA-MNPs-*d*. The yellow bar stands for 1 cm. (b). Tumor volume (left) and survival rate (right) of the nude mice as a function of time (day) upon the treatment by (a) OA-MNPs-*a*, (b) OA-MNPs-*b*, (c) OA-MNPs-*c*, and (d) OA-MNPs-*d*. The animal test is plotted with the significance *p* < 0.05 labeled by symbol *, 0.001 < *p* < 0.01 labeled by symbol **, and *p* < 0.001 labeled by symbol ***. The standard deviation for a and b is n = 6. (c). Prussian blue staining of tumor tissue upon the treatment by (a) OA-MNPs-*a*, (b) OA-MNPs-*b*, (c) OA-MNPs-*c*, and (d) OA-MNPs-*d*. The yellow bar stands for 15 μm. The white arrow means that the cells are treated by the OA-MNPs, and the black arrows mean that the tumor tissue and liver tissue. (d). Anti-ssDNA analysis of tumor tissue upon the treatment by (a) OA-MNPs-*a*, (b) OA-MNPs-*b*, (c) OA-MNPs-*c*, and (d) OA-MNPs-*d*. The yellow bar stands for 15 μm. The black arrows mean that the tumor tissue and liver tissue. (e). Magnetic hysteresis loops of tumor tissues upon the treatment by (a) OA-MNPs-*a*, (b) OA-MNPs-*b*, (c) OA-MNPs-*c*, and (d) OA-MNPs-*d*. (f). Serological and toxicology test of nude mice, including the density of platelet (left), the density of white blood cell (middle), and the density of red blood cell (right), upon the treatment by (a) OA-MNPs-*a*, (b) OA-MNPs-*b*, (c) OA-MNPs-*c*, and (d) OA-MNPs-*d*. The serological and toxicology test are plotted with the significance *p* < 0.05 labeled by symbol *, 0.001 < *p* < 0.01 labeled by symbol **. The bars stand for the standard deviations (n = 3).

The animal results show that the OA-MNPs-*d* injection was extremely efficacious in the tumor reduction in comparison with the OA-MNPs-*a*, -*b*, and -*c* (Figs. 3a and 3b). The tumor volume as a function of surviving time (day) increases for the animals treated by the OA-MNPs-*a* and -*b*, but decreasing for those treated with the OA-MNPs-*c* and -*d*. The effect of the OA-MNPs-*d* treatment is particularly strong, characterized by the mean volume of the tumor of 2687.67 ± 49.22 mm^3^ (n = 6) on the 13rd day, significantly smaller than all the other groups. Three of the six animals treated by the OA-MNPs-*d* survived for 23 days. An obvious reason for this enhanced efficacy is that the OA-MNPs-*d* bind more folate to the folate receptor on the membrane of the HeLa cells. If the targeted OA-MNPs are internalized (endocytosis) after the binding to the folate receptor, as demonstrated here, the subsequent intracellular delivery of drugs may contribute significantly to the cytotoxicity and enhanced efficacy of this group in tumor reduction.

We then present the obtained results from the Prussian blue staining. The pictures on the excised tumors at the injection sites and the liver tissue are shown Fig. 3c. The blue color for multi-target MNPs and the red color for tumor cell nuclear can be seen. However, according to the cell morphology, the Prussian blue staining of the tumor tissue for the OA-MNPs-*d* treated group shows that the tumor cell apoptosis is more remarkable than the others (Fig. 3c (d1)), while nearly no multi-target MNPs can be found in the liver tissue (Fig. 3c (d2)).

The pictures of histological staining on the excised tumors at the injection sites and the liver tissue are given in Fig. 3d. The tumor reduction is confirmed by the anti-ssDNA staining. In both the OA-MNPs-*c* and -*d* treated groups, the anti-ssDNA staining of the median tumors at the end point demonstrates that the apoptosis behavior is consistent with the variation of tumor volume, while the apoptosis is more remarkable (the nuclear chromatin apparently condenses to the deep degree, forming chromatin blocks) in the OA-MNPs-*d* treated group than that in the others (Fig. 3d (d1)). However, according to the cell morphology without chromatin condensation, the anti-ssDNA staining of the liver tissue for the OA-MNPs-*d* treated group shows that the drug toxicity is relatively less than the other groups (Fig. 3d (d2)).

In Fig. 3e ((a), (b), (c), (d)) are plotted the magnetic hysteresis of tumor tissues measured at 298 K. Given the fact that the injected drugs are very small in mass weight, and given the fact that no SQUID signals from the samples without any MNPs injection can be detected, the SQUID signals shown in Fig. 3e are sufficient for demonstrating the existence of MNPs in these samples. The saturation magnetizations treated by the OA-MNPs-*a*, -*b*, -*c*, and -*d* are 0.00215, 0.00265, 0.00388, and 0.00398 emu/g, respectively. Also in the same state, the saturation magnetization intensity of oleic acid coated Fe_3_O_4_ is 70.7 emu/g. It can be estimated that the tumor tissue per gram contains 30.4 μg OA-MNPs-*a*, 37.47 μg OA-MNPs-*b*, 54.9 μg OA-MNPs-*c*, and 56.3 μg OA-MNPs-*d*, respectively.

Moreover, we assess the toxicity of each group *in vivo* by counting the white blood cells (WBC), blood platelets (PLT), and red blood cells (RBC). The assessment of the WBC, PLT, and RBC counts in the OA-MNPs-*d* treated group at the 13^th^ day point reveals the low evidence with leukopenia or associated toxicities (Fig. 3f). One explanation for this significantly enhanced efficacy and relatively weak changes of the WBC, PLT, and RBC counts is that the OA-MNPs-*d* can be more efficiently internalized into tumor cells than the other three kinds of OA-MNPs, with subsequent internalization of the drug. The latter may locate in the extracellular space, causing systemic absorption and distribution, thus increased toxicity and decreased efficacy.

### Cell death molecular mechanism *in vitro*

**Figure 4:**
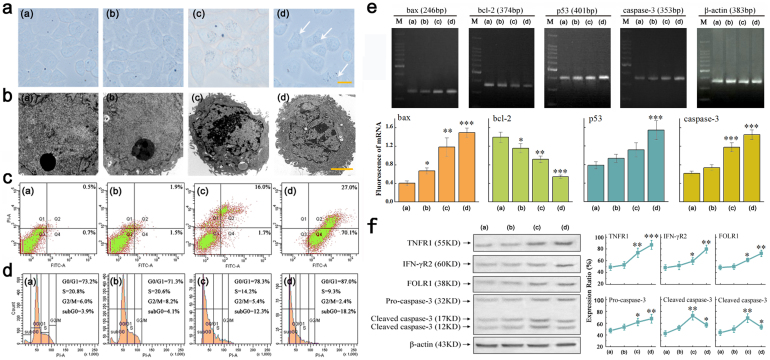
Measured efficacy data *in vitro*. HeLa cells were treated with (a) OA-MNPs-*a*, (b) OA-MNPs-*b*, (c) OA-MNPs-*c*, and (d) OA-MNPs-*d* for 48 hours. (a) and (b). Cells morphology as evaluated by light microscopy (Yellow bars, 15 μm) and transmission electron microscopy (Yellow bars, 5 μm). The white arrow means that the cells are uptaked by the OA-MNPs. Only one of three representative experiments is shown here. (c). Measured cell mortalities data by flow cytometry (n = 3). (d). Measured cell cycle data by flow cytometry (n = 3). (e). Measured mRNA expression of p53, Bax, Bcl-2 and caspase-3 by PCR and RTPCR analysis. In the PCR analysis, data are representative of three experiments. In the RTPCR anaylsis, the relative levels of p53, Bax, Bcl-2 and caspase-3 are plotted with the significance *p* < 0.05 labeled by symbol *, 0.001<*p*<0.01 labeled by symbol **, and *p*<0.001 labeled by symbol ***, in comparison with the OA-MNPs-a group. The bars stand for the standard deviations (n = 3). (f). Measured protein expression data of TNFR1, IFNR2, FOLR1, and caspase-3 (cleaved caspase-3) by western blotting. Blots were re-probed for β-actin and used as control for equal loading of proteins. Protein expression determined using BandScan software. The relative levels are plotted with the significance *p* < 0.05 labeled by symbol *, 0.001 < *p* < 0.01 labeled by symbol **, and *p* < 0.001 labeled by symbol ***, in comparison with the OA-MNPs-a group. The bars stand for the standard deviations (n = 3).

Although the difference between the OA-MNPs-*c* and OA-MNPs-*d* in terms of their inhibition effect on the HeLa cells is not very remarkable, the performance of the OA-MNPs-*d* is indeed better. Fig. 4a((c), (d)) and Fig. 4b((c), (d)) indicate that the cells are in the stage of programmed cell death, with the nuclear chromatin apparently condensed to the deep degree with chromatin blocks. Moreover, the existence of a big number of integrity, smaller and even multinuclear cells, indicates that the inhibition upon the treatment by the OA-MNPs-*d* is more significant than that treated by OA-MNPs-*c* in promoting the programmed cell death. Fig. 4a shows that the MNPs are inside the HeLa cells and on the cells' surfaces.

At the same time, the treatments by the OA-MNPs-*c* and -*d* lead to cell mortality rates of 17.7% and 97.1%, respectively, while the rates for the OA-MNPs-*a* and -*b* are only 1.2% and 3.4%, respectively, as shown in Fig. 4c, demonstrating the superior performance of the OA-MNPs-*d* with respect to others. Fig. 4d presents the HeLa cell DNA content histograms. While the control groups (OA-MNPs-*a* and -*b*) show a shoulder in the S phase, the number of cells is much lower in the two cases treated by the OA-MNPs-*c* and -*d* than those in the controls, noting the remarkable change in the G_1_ phase for the two cases. This demonstrates that the cells treated by the OA-MNPs-*c* and -*d* are mainly arrested in the G_1_ phase, delaying the G_1_/S phase conversion.

We subsequently investigate the p53, Bax, Bcl-2 or caspase-3 mRNA levels in various HeLa cell groups, treated respectively by the four kinds of OA-MNPs for 48 hours. The obtained results are summarized in Fig. 4e. Indeed, the OA-MNPs-*d* cause remarkable enhancement of p53, Bax or caspase-3 mRNA levels. No such enhancement for the OA-MNPs-*c* is observed. These results indicate that the OA-MNPs-*d* may trigger the PCD by up-regulating the mRNA expression of p53, Bax or caspase-3, but not that of apoptosis suppressor gene Bcl-2. Furthermore, the fact that a brighter band of p53, Bax or caspase-3 is potently induced by the OA-MNPs-*d* suggests that the OA-MNPs-*d* may trigger the PCD by up-regulating the mRNA expression of p53, Bax or caspase-3.

Finally, we analyze the activities of the TNFR1 (TNF-α receptor), IFNR2 (IFN-γ receptor), FOLR1 (folate receptor), and caspase-3 (or cleaved caspase-3) in response to the treatments by the four OA-MNPs, respectively (Fig. 4f). The TNFR1, IFNR2, and FOLR1 expressions at 55 kDa, 60 kDa and 38 kDa show more significant up-regulating for the OA-MNPs-*d* case at 48 hours than the OA-MNPs-*c* cases at the same time. The TNFR1 and IFNR2 data suggest that the TNF-α and IFN-γ control capability on the outer membrane of the HeLa cells induced by the OA-MNPs-*d* may be more significantly activated. Similarly, big differences in the levels of FOLR1 expression for the four OA-MNPs are observed. The OA-MNPs-*d* treatment induces more up-regulating of the level of FOLR1 (38 kDa) expression at 48 h, which suggests more folic acid receptor expression on the membrane of the HeLa cells. However, interestingly, comparing with OA-MNPs-*d*, the OA-MNPs-*c* treatment induces the more up-regulating of the level of cleaved (active) caspase-3 (17 kDa and 12 kDa) expression at 48 h, respectively.

## Discussion

In recent years, for effective drug delivery, many incorporation or loading methods for different drugs onto nanocarriers have been developed. For example, direct parcel adsorption method (vacuum/ultrasound-assisted nanocasting)^40^,^41^,^42^,^43^, emulsion/solvent evaporation^44^,^45^, polymerization reactions^46^,^47^, chemical bonds^19^ (such as amide bond, pH-sensitive hydrazone bonds), etc. However, direct parcel method often leads to unclose and unstable adsorption. Emulsion/solvent evaporation method is difficult to control the stability and dispersion for generating the nanoparticles. Polymerization reactions method may lead to incomplete combination. Chemical bonding method often means complex chemical reaction, requiring a catalyst and proper reaction temperature. Therefore, successful synthesis of highly efficient nanocarriers for drug delivery remains challenging so far.

To circumvent these problems, the strategy of the present work is to take advantage of the character and performance of the MNPs functionalized with folic acid (small particle size, large specific surface, coupling of high capacity, magnetic response, superparamagnetic, and folate receptor mediated endocytosis). Their nontoxic nature and potential for nanoparticle synthesis, coupled with high loadings of different drugs following cell biology mechanism by simple but high effective photo-immobilization approach, make them ideal candidates for drug-delivery nanocarriers.

We compare two photo-immobilization methods (LPI & SPI) to prepare nanoparticles. Our results show that the LPI is superior to the SPI for the preparation of the multi-target MNP drug carrier in high drug molecules loading and low molecular skeleton change. Our biomedical characterizations also indicate that the LPI is superior to the SPI. Upon the treatment by the OA-MNPs-*d* (using the LPI scheme), one observes both the enhanced efficacy in tumor reduction and the decreased drug toxicity *in vivo*. Moreover, the OA-MNPs-*d* cause the remarkable changes in the cell morphology and mortality, and the specific cell cycle G_1_ arrest as well as the enhancement of p53, Bax or caspase-3 mRNA levels and TNFR1, IFNR2, FOLR1 protein activities *in vitro*.

However, surprisingly, OA-MNPs-*d* does not induce up-regulating of the level of active caspase-3 expression at 48 h. This fact reveals that an additional caspase-independent cell death pathway (i.e. mitotic catastrophe, cell morphology can be shown in Fig. 4b (d)), in concert with caspase-dependent apoptosis pathway, may cause more remarkable death of HeLa for the OA-MNPs-*d* case.

Generally, mitotic catastrophe can be induced by DNA damage, disruption of mitotic spindles, prolonged growth arrest, or inhibition of the cyclin-dependent kinase, CHK1, or Aurora kinases. Mitotic abnormalities lead to the formation of interphase cells with multiple micronuclei, followed by caspase-independent cell death. Because a kind of different cell death pathway is activated, inducing mitotic catastrophe is a valuable and novel mechanism for overcoming drug resistance^48^,^49^,^50^.

The signal transduction promotion of HeLa cells by the polystyrene co-immobilized with TNF-α plus IFN-γ was reported in our earlier works. It is suggested that the co-immobilized TNF-α plus IFN-γ promotes the activation of some key markers in response to IFN-γ, and the binding of the co-immobilized TNF-α plus IFN-γ with the novel TNF-α receptors results in enhanced programmed death in HeLa cells (apoptosis and apoptosis-like PCD)^37^,^38^,^39^. In the present work the up-regulating of protein expression of TNFR1 and IFNR2 and the up-regulating of the mRNA expression of p53, Bax or caspase-3 suggest that the TNF-α and IFN-γ control capability on the outer membrane of the HeLa cells induced by the OA-MNPs-*d* may be more significantly activated.

Moreover, our present results reveal that the LPI strategy also leads to more folic acid receptor (FOLR1) expression on the membrane of the HeLa cells for OA-MNPs-*d* case, which suggest more OA-MNPs with photo-immobilized DOX entering into the HeLa cells. Since the nuclear DNA is the main subcellular site of DOX action for its antitumor activity, we argue that the LPI method contributes to the inner cell death mechanism control for efficient cancer treatment. Further investigations are definitely deserved.

In summary, we have developed a novel inner/outer control multi-target MNP drug carrier for target delivery by means of the liquid photo-immobilization technique. We have demonstrated the high *in vivo* and *in vitro* efficacy of this liquid immobilized OA-MNP drug carrier in treating the human cervical cancer HeLa cells. We have demonstrated that this liquid photo-immobilization technique as a generalized approach to synthesize various nanoparticle drug carriers against other important human cancers is very promising, although deep investigations to elucidate the cell biology molecular mechanism associated with these novel and powerful magnetic nanoparticle drug carriers are needed.

## Methods

### Synthesis of AzPhTNF-α, AzPhIFN-γ, AzPhDOX, and AzPhFOL

The preparation of photoactive TNF-α, IFN-γ, DOX, and FOL (abbreviated as AzPhTNF-α, AzPhIFN-γ, AzPhDOX, and AzPhFOL, respectively) was described previously and a schematic drawing of the procedure is given in Fig. 1a as an example^31^. All the treatments were carried out in the dark. In a separate sequence, the TNF-α (CELSTAR Bio-Pharmaceutical Co. Ltd., Shanghai) in 30 μg/0.002 μmol and the IFN-γ (The Third Affiliated Hospital of Sun Yat-Sen University, Roche Company, Shanghai) in 30 μg/0.002 μmol were separately added into two vessels each with 3 ml DMF/PBS (4:1) (Sigma) which contains 61.5 μg/0.2 μmol *N*-(4-azidobenzoyloxy) succinimide (Santa Cruz Biotechnology, USA). The DOX (Main Luck Pharmaceuticals Co. Ltd., Shenzhen) in 10 μg/0.017 μmol was added into a vessel with 3 ml DMF/PBS (4:1) (Sigma) which contains 442 μg/1.7 μmol *N*-(4-azidobenzoyloxy) succinimide (Santa Cruz Biotechnology, USA). The FOL (Xiang Bo Bio-Technology co., Ltd., Guangzhou) in 10 μg/0.023 μmol was added into a vessel with 392.38 μg/2.3 μmol 4-Azidoaniline hydrochloride (Sigma, USA). Then they were separately frozen down to 4°C and magnetically stirred for 48 h. After the full reactions, these TNF-α, IFN-γ, DOX, and FOL derivatives were respectively purified in dialysis membrane (Milipore Molecut II, 500) for 72 hours. The as-prepared photoactive AzPhTNF-α, AzPhIFN-γ, AzPhDOX, and AzPhFOL were stored at 4°C for subsequent usages.

### Co-immobilized OA-MNPs with AzPhTNF-α, AzPhIFN-γ, AzPhDOX, and AzPhFOL

The oleic acid coated MNPs (OA-MNPs) were synthesized in our laboratory, following the procedure reported in literature^7^,^8^,^9^. For the solid photo-immobilization, the as-prepared AzPhTNF-α, AzPhIFN-γ, AzPhDOX, AzPhFOL (all at an initial dose of 10 ng), and OA-MNPs (at an initial dose of 1000 ng), were separately added into the phosphate buffered solution (PBS, pH7.4) for stabilizing for 48 hours, followed by the subsequent treatments: the solution was vibrated sufficiently and then dried at 4°C in the air. Subsequently, the OA-MNPs mixed with the AzPhTNF-α, AzPhIFN-γ, AzPhDOX, and AzPhFOL (be spread onto a glass plate with a layer of ~2.0 mm in thickness) were irradiated with the UV lamp (125 W) for 5–10 min at the distance of 10 cm. For the liquid photo-immobilization, the as-prepared AzPhTNF-α, AzPhIFN-γ, AzPhDOX, and AzPhFOL (all at an initial dose of 10 ng), and the OA-MNPs (at an initial dose of 1000 ng) were separately added into the PBS solution for stabilizing for 48 hours, followed by the subsequent treatments: the PBS solution with the OA-MNPs plus AzPhTNF-α, AzPhIFN-γ, AzPhDOX, and AzPhFOL were transferred to a culture vessel and irradiated with the UV lamp (125 W) for 5–60 min at the distance of 10 cm, by keeping the vessel rotating at the speed of 100 rpm/min. Given the feature of highly active azido group, the AzPhTNF-α, AzPhIFN-γ, AzPhDOX, and AzPhFOL were immobilized on the surface of the OA-MNPs (in prior to this procedure, all the treatments were prepared in the dark). The above as-prepared magnetic nanoparticle drug carriers by the two photo-immobilization methods were thoroughly purified with PBS solution in dialysis membrane (Milipore Molecut II, 500) for 72 hours and then stored at 4°C.

### Microscopic surface and functional group analysis

After the photo-immobilization procedure, it is critical to check whether the photoactive AzPhTNF-α, AzPhIFN-γ, AzPhDOX, and AzPhFOL are immobilized onto the surface of the OA-MNPs or not. Fig. 2a presents the scanning electron microscopy images of the four powder samples (SEM, JSM-7600F, Japan). In addition, the surface morphology was also investigated by atomic force microscopy (AFM, Cypher, Asylum Research, USA).The particle size distribution was evaluated by the dynamic light scattering (DLS) using the Zetasizer Nano-ZS90, Malvern Instruments Ltd, England. The Fourier transform infrared spectroscopy (FTIR) and Raman spectroscopy were performed using the TENSOR27, Bruker, Germany and LabRAM Aramis, France. The difference in the surface morphology and molecular structure for the four samples (the OA-MNPs-*a*: the mixture of OA-MNPs and photoactive drug molecules (TNF-α, IFN-γ, DOX and FOL) in prior to photo-immobilization; the OA-MNPs-*b*: the OA-MNPs taken from the solid photo-immobilized powder after removing the top layer of ~1.0 mm in thickness; the OA-MNPs-*c*: the solid-immobilized powder without removing the top layer; and the OA-MNPs-*d*: the liquid photo-immobilized powder) indicates that the samples OA-MNPs-*c* and OA-MNPs-*d* did have the photo-immobilized AzPhTNF-α, AzPhIFN-γ, AzPhDOX, and AzPhFOL on the surface of the OA-MNPs, as shown in Fig. 2.

### Grafting ratios measurement

The TNF-α and IFN-γ were diluted with PBS to be the standard solution in gradient concentrations of 0.5 μg/ml, 1 μg/ml, 1.5 μg/ml, 2 μg/ml, 2.5 μg/ml. Also DOX and FOL were diluted with PBS to be the standard solution in gradient concentrations of 10 μg/ml, 20 μg/ml, 30 μg/ml, 40 μg/ml, 50 μg/ml. Then UV spectrophotometry was used to detect at the wavelength of 230 nm, 280 nm, 233 nm, 350 nm, respectively. The absorption value of PBS phosphate buffer solution was the control. The absorption value was detected to draw standard curve. The regression equations are C = 0.005A + 0.0006 (R2 = 0.9998), C = 0.0803A + 0.0026 (R2 = 0.9992), C = 0.0503A + 0.0016 (R2 = 0.9992), and C = 0.0589A + 0.0057 (R2 = 0.9994), respectively. The OA-MNPs-*a*, OA-MNPs-*b*, OA-MNPs-*c*, and OA-MNPs-*d* were dissolved in PBS. We used the dialysis or ultrafiltration to remove drug which was not be grafted. The absorbance values were measured in the corresponding wavelength, and the grafting ratios (Fig. 2e) were calculated in the regression equations of different drugs (TNF-α, IFN-γ, DOX and FOL).

### Animal studies

Animals (nude mice) were purchased from the Experimental Animal Center of Guangdong Province and cared for under the supervision of the Experimental Animal Center of Sun Yat-sen University. Xenograph flank tumors were induced in 4-week old BALB/c nude mice by subcutaneous (s.c.) injection of 1 × 10^7^ HeLa cells/nude mouse. After 3 weeks, when tumors had reached ~3000 mm^3^, mice were divided into four groups of six mice, minimizing weight and tumor size differences. Tumor-bearing nude mice were treated by intravenous injection of the four OA-MNPs samples (OA-MNPs-*a*, OA-MNPs-*b*, OA-MNPs-*c*, and OA-MNPs-*d*) every other day. After the dosing, the mice were monitored for implanted tumor size daily for 3 weeks and every 3 days thereafter. The length and width of the tumors were measured by digital calipers. Tumor volume was calculated by the following formula: ((width × length)/2)^2^. Mice were monitored for a maximum of 24 days. For animals put to death by dislocation of infra-cervical spine, the tumor size at the time of death was used for the purpose of mean tumor size calculation, as shown in Figs. 3a and b.

### Prussian blue staining

Paraffin embedded tissue was placed in water. Hydrochloric acid and potassium ferrocyanide solution in 1:1 volume mixing ratio was freshly prepared and used to be working solution. Sections were immersed in working solution for standing 10–30 minutes. After the reaction, sections were immersed in distilled water for 3 minutes and 3 times, and then rinsed thoroughly. Nuclear fast red solution was dropped in, covering the specimen for 5–10 minutes. After the reaction, sections were immersed in distilled water for 3 minutes and 3 times. And then sections were immersed in 95% alcohol and xylene to dehydrate for 2 minutes. The mounting medium was dropped onto the sections and covered with coverslip. The distribution of OA-MNPs with Prussian blue staining was observed under the microscope, as shown in Fig. 3c.

### Anti-ssDNA analysis

Tumor tissue was fixed in 4% paraformaldehyde in 0.1 M sodium phosphate buffer, pH 7.4, for 12 h at 4°C and then embedded in paraffin wax according to standard procedures. Sections were carefully dewaxed and rehydrated in phosphate-buffered saline (PBS) and incubated in ice-cold HCl for 15 min and incubated in 90°C PBS supplemented with 0.2% Triton X-100 and 5 mM MgCl_2_ for 6 min. After that, sections were transferred into ice-cold PBS for 10 min and pretreated with 0.03% hydrogen peroxide in methanol to inhibit endogenous peroxidase activity. Immunoreactivity was detected by the avidin-biotin complex (ABC) method. After treatment with normal 10% goat serum for 12 min, sections were incubated overnight with antibody (ssDNA, diluted 1:60 in PBS) at 4°C. The antigen-antibody reactions were detected by incubation with biotin-labeled goat anti-mouse IgG for the ssDNA antibody (TNT-3; Abcam Inc, USA). The enzyme reaction was developed with a mixture of diaminobenzidine (DAB) and H_2_O_2_ in 0.05 M Tris-HCl buffer (pH 7.6). Also, sections were counterstained with hematoxylin and mounted with Neutral balsam. Finally, the morphology of tumor tissue was observed using a light microscope, as shown in Fig. 3d.

### Magnetic measurement

Through the high temperature heating, the tumor tissue was turned into a powder state. Then the magnetic measurements were performed by using a superconducting quantum interference device (SQUID, Quantum Design Inc., USA) at room temperature. As the references, the samples without injection of these MNPs were submitted to the magnetization measurements, and no reliable magnetic signals can be detected.

### Serological detection

We used the sterilized scissors to take blood samples from the tails of tumor-bearing nude mice treated by intravenous injection of four OA-MNPs groups (the OA-MNPs-*a*, OA-MNPs-*b*, OA-MNPs-*c*, and OA-MNPs-*d*) every other day. Before this procedure, a hair dryer blowing was used for tumor-bearing nude mice to get the blood circulation faster. For each animal, 1.5 ml blood was obtained and preserved in EP tube adding anticoagulants. When taking blood was completed, all the samples were sent to the south hospital (Guangzhou, China) to carry out the serum detection including the white blood cells (WBC), blood platelets (PLT), and red blood cells (RBC). The serological detection results of four OA-MNPs groups were shown in Fig. 3f.

### Cell morphology

Human cervical cancer HeLa cells were seeded at 1 × 10^5^ cells/ml into the 24-well PSt culture plate placing the four OA-MNPs groups for 48 h. After the stimulation, the morphology and inner structure of the HeLa cells were characterized by light microscopy (Nikon) and transmission electron microscopy (TEM, Philips EM400), as shown in Figs. 4a and b.

### Cell cycle arrest

Moreover, by the 48 h stimulation, the floating and adherent cells were combined and the cell viability is determined using the trypan blue dye exclusion method. For the DNA content probing, 1 × 10^6^ cells were fixed and permeabilized in 70% ethanol, washed in phosphate-buffered saline (PBS, pH7.4) treated with RNase (40 U/μl) and stained with propidium iodide (PI) (50 μg/ml). The flow cytometric analysis was performed, as shown in Fig. 4c.

### Cell mortality

Then after 48 h stimulation, the HeLa cells were collected and washed with Dulbecco PBS, re-suspended in binding buffer, and incubated with Annexin V-FITC for 15 min at room temperature in the dark. After the centrifugation, Annexin V-FITC was removed and the HeLa cells were stained with PI in binding buffer. At last, the HeLa cells were analyzed immediately by flow cytometry (Becton Dickinson, FACSC alibur, San Jose, CA) using the Cell Quest Program, as shown in Fig. 4d.

### Real-time fluorescent quantitative PCR and PCR analysis

The relative levels of p53, Bax, Bcl-2 or caspase-3 mRNA in HeLa were examined by RT-PCR (SYBR Green) (ABI 3900, High-Throughput DNA/RNA Synthesizer; ABI 9700, PCR instrument; ABI 7500, fluorescent quantitative PCR instrument; Applied Biosystems, USA), as shown in Fig. 4D. The HeLa cells were treated by four OA-MNPs groups (the OA-MNPs-*a*, OA-MNPs-*b*, OA-MNPs-*c*, and OA-MNPs-*d*) for 48 hours. The following primers of p53, Bax, Bcl-2 or caspase-3 designed with Primer Express 2.0 Software were used for the PCR step: (1) p53, Bax, Bcl-2 or caspase-3 sense, 5′-CCGAGTGGAAGGAAATTTGC-3′, 5′-CATGTTT TCTGACGGCAACTTC-3′, 5′-TGGGATGCCTTTGTGGAACT-3′ or 5′-TACCAGTGGA GGCCGACTTC-3′; (2) p53, Bax, Bcl-2 or caspase-3 antisense, 5′-AGTCAGAGCCAA CCTCAGGC-3′, 5′-AGGGCCTTGAGCACCAGTTT-3′, 5′-GAGACAGCCAGGAGAAAT CAAAC-3′ or 5′-CAAAGCGACTGGATGAACCA-3′; (3) β-actin sense, 5′-GCGCGG CTACAGCTTCA-3′; and (4) β-actin antisense, 5′-TCTCCTTAATGTCACGCACGAT-3′. The gene levels were normalized by re-probing the blots with antibody to β-actin (Boster Biological Technology Co., Ltd., China), as shown in Fig. 4e.

### Western blotting analysis

Finally, the HeLa cells stimulated with the same amount of time were lysed in extraction buffer (10 mM Tris (pH7.4), 150 mM NaCl, 1%Triton x-100, 5 mM EDTA (pH8.0)). The protein samples were separated by SDS-PAGE (12%) and electro-transferred onto a NC (nitrocellulose) membrane (Boster Biotechnology Co., Ltd., China). The expression of proteins was obtained using antibodies to TNFR1, IFNR2, FOLR1, and caspase-3 (Boster Biotechnology Co., Ltd., China). The blots were incubated with appropriate secondary antibodies conjugated to alkaline phosphatase (AP) peroxidase (Boster Biological Technology Co., Ltd., China). The protein levels are normalized by re-probing the blots with antibody to β-actin (Boster Biological Technology Co., Ltd., China), as shown in Fig. 4f.

### Statistical analysis

Statistical results were obtained using the statistical software SPSS17.0. ANOVA (one way analysis of variance) was used to analyze statistical differences between groups under different conditions, and the Student's t-test was performed. The statistical significance *P* < 0.05 is considered statistically significant.

## Author Contributions

Y.Q.G. and J.M.L. designed experiments, conceived the communication strategy, analysed results and wrote the manuscript; Z.Z., S.Q.N. and Z.H. carried out experiments and analysed results. Z.B.L. contributed reagents and technical expertise.

## Supplementary Material

Supplementary Information Supplementary-to-manu
