# Supplementary material for: Powerful inner/outer controlled multi-target magnetic nanoparticle drug carrier prepared by liquid photo-immobilization
Source: Sci Rep. 2014 May 21;4:4990. doi: 10.1038/srep04990 (PMC4028896; doi:10.1038/srep04990)
Supplement: Supplementary Information — Supplementary-to-manu [file srep04990-s1.doc]

***Supplementary materials for manuscript***

**Powerful inner/outer controlled multi-target magnetic nanoparticle drug carrier prepared by liquid photo-immobilization**

Yan-Qing Guan, Zhe Zheng^∆^, Zheng Huang, Zhibin Li, and Shuiqin Niu^∆^

Institute for Advanced Materials and School of Life Science, South China Normal University, Guangzhou 510631, China

Jun-Ming Liu[[1]](#footnote-2)*

Laboratory of Solid State Microstructures, Nanjing University, Nanjing 210093, China

Institute for Advanced Materials and Laboratory of Quantum Engineering and Quantum Materials, South China Normal University, Guangzhou 510631, China

***Experimental Section***

*1. Optimization of the two photo-immobilizations*

For optimizing the immobilization parameters in both the solid and liquid photo-immobilization procedures, the Fourier transform infrared spectroscopy (FTIR) (TENSOR27, Bruker, Germany) was performed to probe the characteristic peaks. The measured data are summarized in Supplementary-FIG. 1, where detailed description of the samples in one-to-one correspondence with these spectrum curves is given in the Figure Caption. It is obviously revealed that the peaks from the aromatic hydrocarbon bonds in the DOX and FOL overlap with the peaks from the amide bonds, due to the photo-immobilization. Thus the characteristic peak at ~1640cm^-1^ for identifying the photoreaction may no longer be used to evidence the photoreaction processes. Nevertheless, by consulting to the fact that the characteristic peaks at ~2837cm^-1^ and ~2903cm^-1^ respectively identifying methyl and methylene disappear but the peak at ~540 cm^-1^ for the Fe-O bond remains unaffected, one may determine the optimizing times for the solid and liquid photo-immobilizations are 10min and 40min, respectively, under the present conditions.

In fact, in case of the solid photo-immobilization, for the top layer of the powder sample, the characteristic peaks at ~2837cm^-1^ and ~2903cm^-1^ for methyl and methylene respectively are no longer available when the UV-irradiation time is over 10min, indicating the photoreaction is already saturated. However, if one removes the top layer of 1.0mm in thickness, these peaks are still available even longer UV-irradiation is performed, suggesting the photoreaction below the top layer of the powder sample is not yet from completed. Thus, we choose 10min to be the optimized time for the solid photo-immobilization.

On the other hand, in case of the liquid photo-immobilization, the two characteristic peaks can still be detected until a photoreaction for 40min, indicating that for the present condition, an UV-irradiation for 40min seems to be sufficient. In the meantime, the peak associated with the Fe-O bonds changes a lot if the photoreaction is longer than 40min, implying a destruction of the surface structure of the OA-MNPs to some extent. Therefore, we choose 40min to be the optimized time for the liquid photo-immobilization.


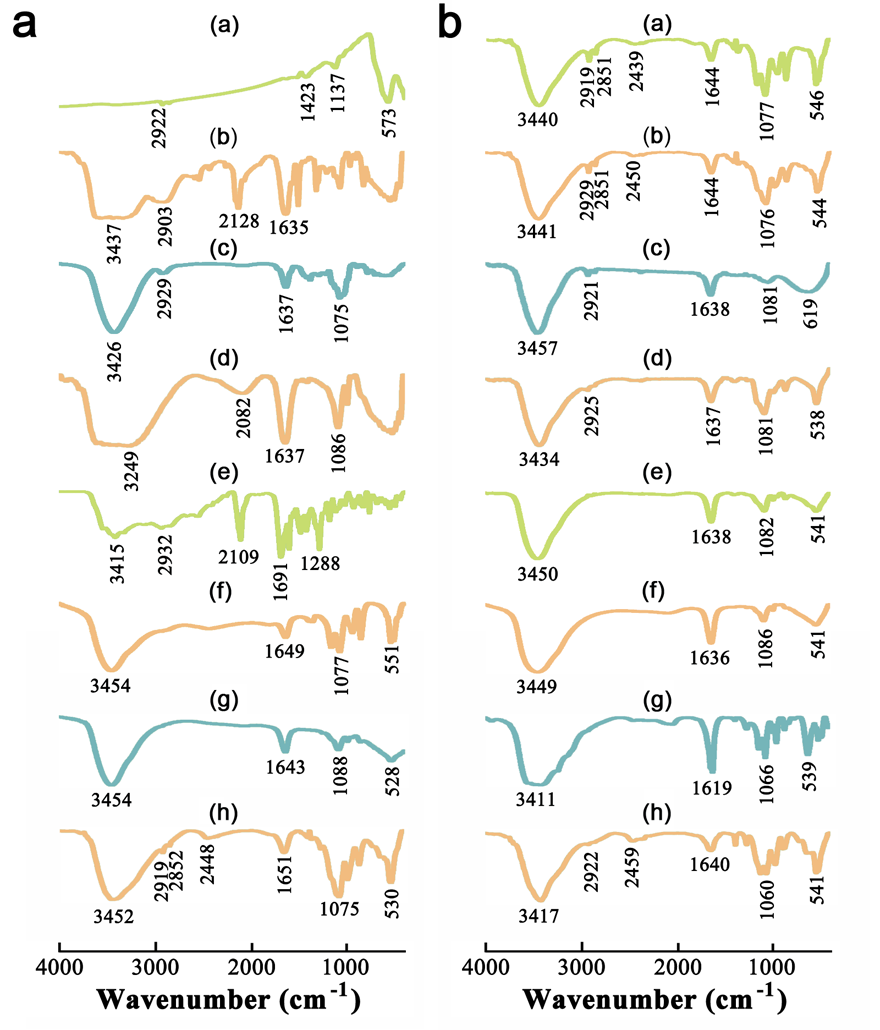


Supplementary-FIG. 1. Measured Fourier transform infrared spectroscopy spectra for various magnetic nanoparticle drug carrier samples. On the column A (left): (a) native OA-MNPs without any drug, (b) *N*-(4-azidobenzoyloxy) succinimide and 4-azidoaniline hydrochloride, (c) DOX, FOL, TNF-α, and IFN-γ without photo-active processing, (d) photoactive DOX, FOL, TNF-α, and IFN-γ, (e) native OA-MNPs plus *N*-(4-azidobenzoyloxy) succinimide, 4-azidoaniline hydrochloride, DOX, FOL, TNF-α, and IFN-γ, without any photo-immobilization, (e)-(h) native OA-MNPs immobilized with DOX, FOL, TNF-α, and IFN-γ for various times by solid photo-immobilization method, where (f) the top layer, 5min; (g) the top layer, 10min; (h) the bottom layer, 10min. On the column B (right): (a) native OA-MNPs plus photoactive DOX, FOL, TNF-α, and IFN-γ, without any photo-immobilization, (b)-(h) native OA-MNPs immobilized with DOX, FOL, TNF-α, and IFN-γ for various times by liquid photo-immobilization method, where (b) 5min, (c) 10min, (d) 20min, (e) 30min, (f) 40min, (g) 50min, and (h) 60min.

*2. Photo-immobilizations of DOX, FOL, TNF-α, and IFN-γ*

For identifying the high effect of native OA-MNPs immobilized with DOX, FOL, TNF-α, and IFN-γ (Co-immobilized 1), we also prepared the native OA-MNPs immobilized with DOX, native OA-MNPs immobilized with TNF-α, native OA-MNPs immobilized with IFN-γ, and the native OA-MNPs immobilized with FOL. Moreover, the native OA-MNPs co-immobilized with DOX, FOL, TNF-α, and IFN-γ (Co-immobilized 2) one by one clearly was also prepared for the comparison with the Co-immobilized-NP1. The schematic diagram is shown in Supplementary- FIG. 2.


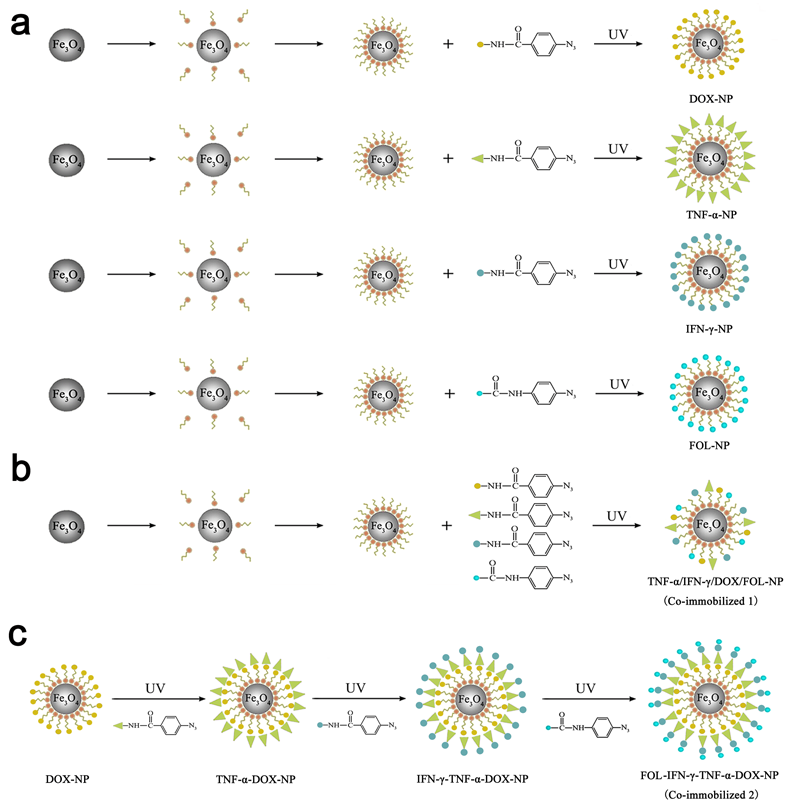


Supplementary-FIG. 2. Schematic drawing of A: photo-immobilization of each of four drugs respectively with the OA-coated particle; B: photo-immobilization of the four drugs with the particle at the same time; C: photo-immobilization of the four drugs subsequently with the particle, where the surface is covered with the FOL molecules and those drugs underneath the surface FOL may no longer be effective.

*3. Cytotoxicity of all the drugs*

The human cervical cancer HeLa, human liver cancer HepG2, and human neuroglioma U251 cells (1×10^5^/well) were seeded in 24-well cell culture polystyrene plates and in 24-well cell culture polystyrene plates treated only with all the drugs for 24 hours at the indicated concentrations. After the treatment, the HeLa, HepG2, and U251 cells were transferred to the 96-well microtiter plates with PBS. Then, MTT (0.5/mg/ml) was added, followed by an incubation at 37^o^C for 2h in a CO_2_ incubator. After a brief centrifugation, the supernatants were carefully removed and DMSO was added. After those insoluble crystals were completely dissolved, the absorbance at 540nm was measured using a Thermomax Microplate Reader (Molecular Devices, Menlo Park, CA).

The results demonstrate that the co-immobilized-1 has the highest inhibit effect on HeLa cells, compared with all the other drugs, as shown in Supplementary-FIG. 3.


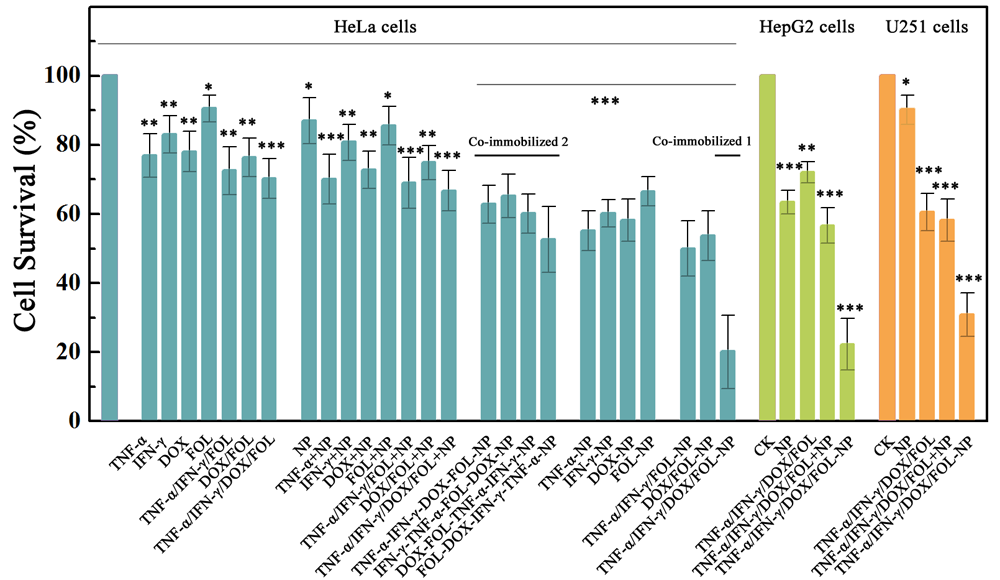


Supplementary FIG.3. Measured HeLa, HepG2 and U251 cell death data with error bars as a function of the dosage of various drugs, as measured by MTT assays. The HeLa, HepG2 and U251 cells were treated for 24 hours with cytokines alone or in combinations with the drugs as individually labeled. The cell survival (%) are plotted with the significance *p*<0.05 labeled by symbol *, 0.001<*p*<0.01 labeled by symbol ** and *p*<0.001 labeled by symbol ***, in comparison with the CK group, with the bars for the standard deviations (n=6).

*4. Animal studies*

Animals (nude mice) were purchased from the Experimental Animal Center of Guangdong Province and cared for under the supervision of the Experimental Animal Center of Sun Yat-sen University. Xenograph flank tumors were induced in 4-week old BALB/c nude mice by subcutaneous (s.c.) injection of 1×10^7^ human liver cancer HepG2 cells/nude mouse. After 3 weeks, when tumors had reached ~180mm^3^, mice were divided into four groups of six mice, minimizing weight and tumor size differences. Tumor-bearing nude mice were treated by intravenous injection of the four OA-MNPs samples (OA-MNPs-*a*, OA-MNPs-*b,* OA-MNPs-*c,* and OA-MNPs-*d*) every other day. After the dosing, the mice were monitored for implanted tumor size daily for 3 weeks and every 3 days thereafter. The length and width of the tumors were measured by digital calipers. Tumor volume was calculated by the following formula: ((width×length)/2)^2^. Mice were monitored for a maximum of 21 days. For animals put to death by dislocation of infra-cervical spine, the tumor size at the time of death was used for the purpose of mean tumor size calculation, as shown in Supplementary-FIG. 4.


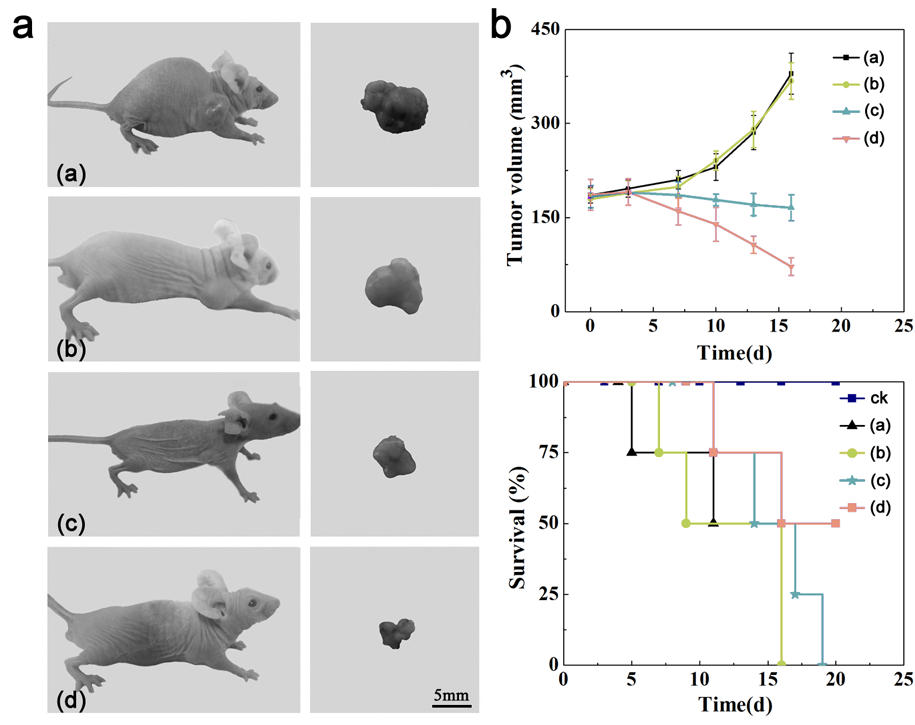


Supplementary-FIG. 4. Measured efficacy data *in vivo*. (A) Photos for tumor-bearing mice treated by the four kinds of OA-MNPs: (a) OA-MNPs-*a*, (b) OA-MNPs-*b*, (c) OA-MNPs-*c*, and (d) OA-MNPs-*d*. The yellow bar stands for 5 mm. (B): Tumor volume (upper) and survival rate (lower) of the nude mice as a function of time (day) upon the treatment by (a) OA-MNPs-*a*, (b) OA-MNPs-*b*, (c) OA-MNPs-*c*, and (d) OA-MNPs-*d*. The standard deviation is n=6.

The animal results show that the OA-MNPs-*d* injection was extremely efficacious in the tumor reduction in comparison with the OA-MNPs-*a* (control group), OA-MNPs-*b*, and OA-MNPs-*c* (Supplementary-FIG. 4A and B). It is shown that the tumor volume as a function of surviving time (day) increases for the animals treated by the OA-MNPs-*a* and OA-MNPs-*b*, but decreasing for those treated with the OA-MNPs-*c* and OA-MNPs-*d*. It is shown that the effect of the OA-MNPs-*d* treatment to HepG2 cells is particularly strong as same as the treatment to HeLa cells.

1. ^*^Correspondent, E-mail: [liujm@nju.edu.cn](mailto:liujm@nju.edu.cn)

   ^∆^These authors contributed equally to this work. [↑](#footnote-ref-2)
